# Dietary intake of bioactive ingredients impacts liver and adipose tissue transcriptomes in a porcine model of prepubertal early obesity

**DOI:** 10.1038/s41598-020-62320-4

**Published:** 2020-03-25

**Authors:** Maria Ballester, Raquel Quintanilla, Francisco J. Ortega, José C. E. Serrano, Anna Cassanyé, Maria Rodríguez-Palmero, José A. Moreno-Muñoz, Manuel Portero-Otin, Joan Tibau

**Affiliations:** 1 Animal Breeding and Genetics Programme, Institute for Research and Technology in Food and Agriculture (IRTA), Torre Marimon, 08140 Caldes de Montbui, Spain; 2 Department of Diabetes, Endocrinology, and Nutrition (UDEN), Institut d’Investigació Biomèdica de Girona (IdIBGi), Girona, Spain; 3 Centro de Investigación Biomédica en Red de la Fisiopatología de la Obesidad y la Nutrición (CIBEROBN), Instituto de Salud Carlos III (ISCIII), Madrid, Spain; 4 Department of Experimental Medicine, University of Lleida-Biomedical Research Institute of Lleida, 25196 Lleida, Spain; 5 Basic Research Department. Ordesa Laboratories, 08830 Barcelona, Spain; 6 Animal Breeding and Genetics Programme, Institute for Research and Technology in Food and Agriculture (IRTA), Finca Camps i Armet, 17121 Monells, Spain

**Keywords:** Gene expression, Obesity

## Abstract

Global prevalence of obesity has increased to epidemic proportions over the past 40 years, with childhood obesity reaching alarming rates. In this study, we determined changes in liver and adipose tissue transcriptomes of a porcine model for prepubertal early obesity induced by a high-calorie diet and supplemented with bioactive ingredients. A total of 43 nine-weeks-old animals distributed in four pens were fed with four different dietary treatments for 10 weeks: a conventional diet; a western-type diet; and a western-type diet with *Bifidobacterium breve* and rice hydrolysate, either adding or not omega-3 fatty acids. Animals fed a western-type diet increased body weight and total fat content and exhibited elevated serum concentrations of cholesterol, whereas animals supplemented with bioactive ingredients showed lower body weight gain and tended to accumulate less fat. An RNA-seq experiment was performed with a total of 20 animals (five per group). Differential expression analyses revealed an increase in lipogenesis, cholesterogenesis and inflammatory processes in animals on the western-type diet while the supplementation with bioactive ingredients induced fatty acid oxidation and cholesterol catabolism, and decreased adipogenesis and inflammation. These results reveal molecular mechanisms underlying the beneficial effects of bioactive ingredient supplementation in an obese pig model.

## Introduction

Obesity is characterized by the excessive accumulation of adipose tissue and its association with many metabolic abnormalities, including (but no limited to) insulin resistance, development of type 2 diabetes, cardiovascular diseases, and cancer^1^. Childhood obesity, favoured by an obesogenic environment, is reaching alarming rates with the major health threat of developing chronic illness as adults. Accordingly, the Commission on Ending Childhood Obesity developed in 2016 a package of recommendations to prevent and treat childhood obesity mainly focused on dietary education and the promotion of physical activity. At the individual level, adjunct approaches such as pharmacotherapy or bariatric surgery have been developed, and the incorporation of bioactive ingredients in the diet has proven effective for the obesity treatment.

Studies in rodent animal models evidenced the anti-obesity beneficial properties of probiotic administration (reviewed in^2^). The supplementation of *Bifidobacterium breve* B-3 in high-fat-diet (HFD)-induced obese mice has been shown to suppress body gain weight and epididymal fat deposition, and to improve serum levels of total cholesterol, glucose and insulin^3^. Expression analyses performed in this mouse model disclosed that the administration of *Bifidobacterium breve* B-3 modified the hepatic expression of genes related to lipid metabolism and response to stress^4^. A reduction in body fat has been also observed in healthy pre-obese individuals receiving *Bifidobacterium breve* B-3, but no significant improvement of blood parameters was found^5^. In partial agreement, studies with rodent models of obesity have demonstrated the anti-obesity effect of n-3 polyunsaturated fatty acids (PUFA). Reduction of fat mass was predominantly observed in mice fed a HFD and supplemented with n-3 PUFA (reviewed in^6^). However, the effects of n-3 PUFA in human obesity remain inconclusive. On the other hand, rice proteins and their hydrolysates added to high fat and/or high cholesterol diets have shown to have anti-obesity and hypocholesterolemic effects through the effective modulation of cholesterol and triglyceride metabolism, and the activity of hepatic genes related to lipid metabolism^7,8^.

Overall, these experimental evidences reinforce the relevance of using bioactive dietetic supplements to prevent and treat obesity. However, as most of these studies were performed in rodent models, the underlying biological mechanisms determining anti-obesity effects are difficult to translate into humans, due to the vast differences between these species at the physiological and metabolic level^9^. In contrast, pigs are excellent models for human obesity and its comorbidities, given their similarity in terms of anatomy, physiology, metabolism, and genetics^9–11^.

To date, a limited number of pig models for childhood obesity have been developed by short term (≤ 12 wk.) dietary interventions of high-fat-regimes in young pigs^9,12,13^. These models seem to develop early features of childhood obesity without developing insulin resistance or metabolic syndrome. In a previous work, a porcine model of prepubertal early obesity induced by a high-calorie diet was characterized at the phenotypic level^14^. Here, we studied the transcriptomic changes in relevant metabolic tissues derived from both feeding a high calorie diet and supplementing with bioactive ingredients, including *Bifidobacterium breve*, rice hydrolysate, and n-3 PUFA, in piglets of this porcine model of prepubertal early obesity. The final aim was to determine the molecular mechanisms and biological processes underlying the beneficial effects of bioactive ingredient supplementation in a porcine model for childhood obesity.

## Results

### Phenotypic differences between piglets feeding different diets and bioactive ingredients

Data regarding growth, feed/energy intake, and biochemical variables in piglets subjected to the four dietary treatments is shown in Table 1. All groups of piglets had similar body weight at the beginning of the experiment (week 9). During the two months of dietary treatment, animals fed a western-type diet without supplementation showed the highest weight gain, reaching a body weight above piglets fed a balanced diet (60.0 kg in T2 vs. 53.3 kg in T1 at week 18). Conversely, piglets supplemented with *Bifidobacterium breve* probiotic and rice hydrolysate (T3 and T4) showed a significantly attenuated weight gain with respect to T2 animals, and reached final weights (51.4 and 50.2 kg at week 18, respectively) close to the control group. In terms of energy intake, all three groups of animals fed western-type diet (T2, T3 and T4) had a calorie ingestion (kcal/day) significantly superior to animals in the control group (T1).

**Table 1:** Significance of dietary treatment effect on piglets weight, growth, feed/calorie intake, fat deposition and biochemical variables, plus least square means (LSmeans) within each treatment: control diet (T1); western-type diet (T2); western-type diet with rice protein and supplemented with probiotic (T3); western-type diet with rice protein and supplemented with probiotic and omega-3 fatty acids (T4).

VARIABLE	SIGN^1^	LSmeans by treatment^2^			
		T1	T2	T3	T4
Body weight at week 9 (kg)	n.s.	16.50^a^	16.70^a^	16.14^a^	16.27^a^
Body weight at week 18 (kg)	*	53.27^a^	60.00^b^	51.36^a^	50.23^a^
Average daily weight gain (kg)	**	0.58^ab^	0.69^b^	0.56^a^	0.54^a^
Average daily feed intake (kg)	**	1.46^a^	1.42^ab^	1.31^ab^	1.26^b^
Average daily calorie intake (kcal)	****	3819.75^a^	5213.93^b^	4876.75^b^	4693.72^b^
Liver weight (gr)	n.s.	1336.18^a^	1518.90^a^	1431.73^a^	1385.45^a^
Weight of flare fat (gr)	****	188.73^a^	405.30^b^	354.27^b^	323.73^b^
Percentage of fat (%)	****	12.66^a^	19.35^b^	17.91^b^	17.74^b^
Percentage of subcutaneous fat (%)	****	8.36^a^	13.20^b^	12.42^b^	12.00^b^
Percentage of intermuscular fat (%)	*	1.27^a^	1.84^b^	1.52^ab^	1.53^ab^
Percentage of flare fat (%)	***	3.03^a^	4.32^b^	3.98^b^	3.89^b^
Total cholesterol in serum (mg/dl)	*	125.90^a^	155.74^ab^	167.16^b^	144.19^ab^
HDL-cholesterol in serum (mg/dl)	***	45.25^a^	61.36^b^	68.91^b^	59.10^b^
LDL-cholesterol in serum (mg/dl)	*	46.87^a^	57.11^b^	51.13^ab^	45.33^a^
Triglycerides in serum (mg/dl)	***	21.67^a^	29.22^ab^	33.42^b^	25.15^a^
Glucose in serum (mg/dl)	n.s.^+^	89.20^a^	108.54^a^	117.80^a^	119.78^a^
Fructosamine in serum (μmol/dl)	n.s.	177.61^a^	152.14^a^	179.50^a^	165.97^a^

^1^SIGN - Significance of dietary treatment effect according to its p-value in a one-way analysis of variance: ****P < 0.0001; *** P< 0.001; **P < 0.01; *P < 0.05; ^+^P < 0.1; n.s. no significant.

^2^Different superindex (a and b) indicates significant differences (P < 0.05) between LSmeans in a Tukey’s HSD test for multiple comparisons.

Besides, important differences regarding body composition were observed between animals in different dietary treatments. The mean percentage of fat estimated through computed tomography (relative fat volume) increased from 12.7% in piglets with a balanced diet to 19.3% in piglets fed the western-type diet. This increase in adiposity derived from high calorie intake applied to all fat depots (i.e. subcutaneous, intermuscular, and flare fat), but particularly to the subcutaneous depot (8.4% in T1 vs 13.2% in T2, P < 0.0001). Regarding the supplementation with *Bifidobacterium breve* probiotic, animals in T3 and T4 also showed an increased adiposity when compared to the control group (T1), but the relative amount of fat in all compartments tended to be lower than in no supplemented piglets (T2).

Serum lipid levels run relatively in parallel to differences observed in fat deposition. Serum concentrations of total cholesterol and both LDL- and HDL-cholesterol, as well as concentrations of triglycerides, were higher in piglets with a high calorie intake, either with or without bioactive supplementation (T2, T3 and T4), than in the control group (T1), although some of the comparisons did not reach statistical significance. To be noted, animals fed on T4 showed significant lower levels of LDL-cholesterol in serum than their T2 counterparts.

### Transcriptomic changes induced by a high calorie diet

To study the effect of a high calorie diet on gene expression, the transcriptomes of liver and two different adipose tissue depots: visceral adipose tissue (VAT) and subcutaneous adipose tissue (SAT), belonging to two groups of piglets fed control (T1; n = 5) and western-type (T2; n = 5) diets were analyzed by RNA-seq. First, we performed principal-component analysis (PCA) of RNA-seq data, identifying an outlier in the control group of SAT (Supplementary Fig. S1) that was removed before further analyses.

**Figure 1:**
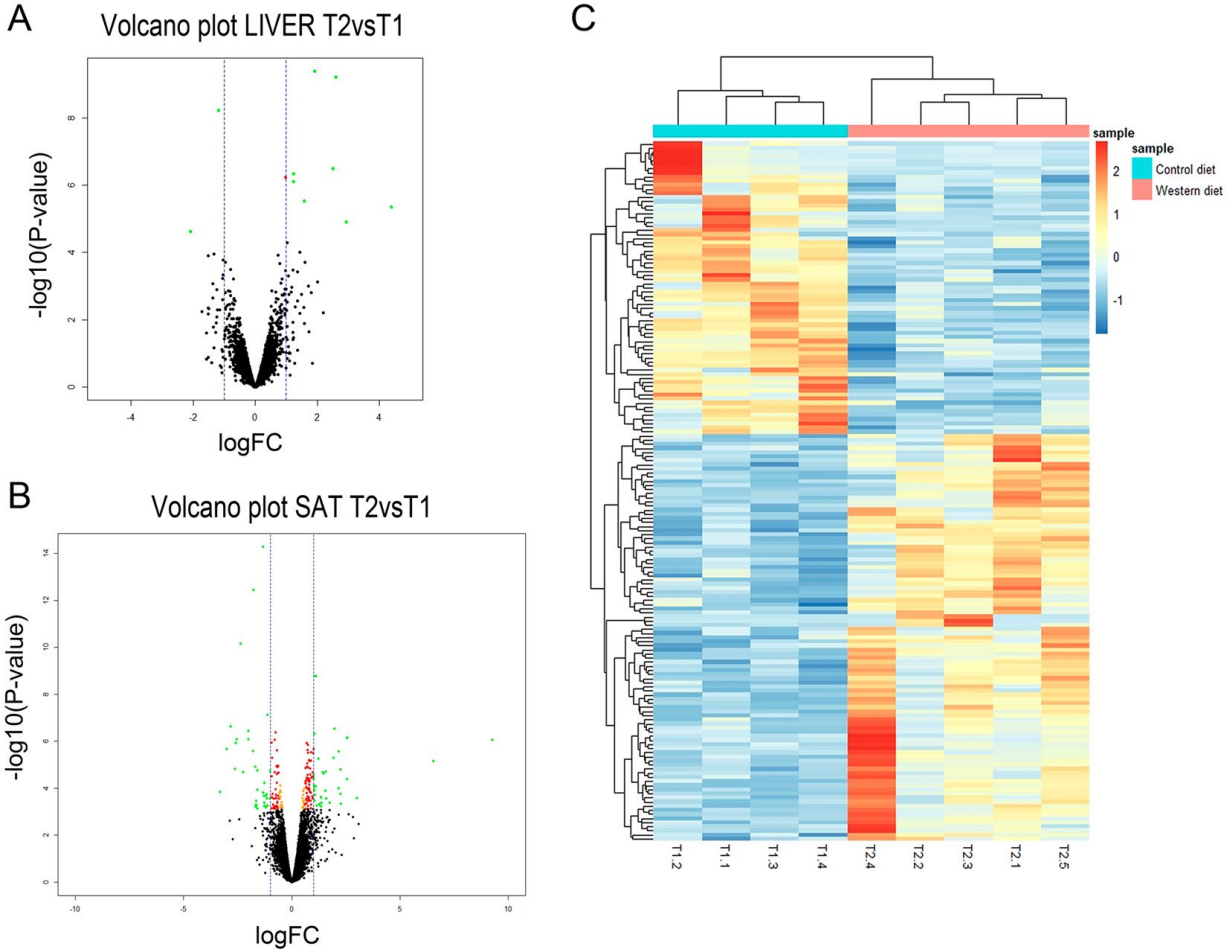
Differentially expressed (DE) genes in liver and subcutaneous adipose tissue (SAT) between T1 and T2 fed animals. Volcano plot displaying DE genes in liver (**A**) and SAT (**B**) between T1 and T2 treatments. The vertical axis (y-axis) corresponds to the −log_10_ (*P*-value), and the horizontal axis (x-axis) displays the log_2_ fold change (logFC) value. Green dots represent DE genes according to a FDR < 0.05 and |logFC| > 1. Red dots represent DE genes according to a FDR < 0.05 and |logFC| > 0.58 and orange dots represent DE genes according to a FDR < 0.05. Positive x-values represent genes upregulated and negative x-values represent genes downregulated in T2vsT1 groups. The vertical dashed lines mark the |logFC| = 1. C) Heatmap of the 171 DE genes between T1 and T2 treatments in SAT. Each row represents a gene (n = 171) and each column represents an individual (n = 9). Line graphs on top of the heatmap show clustering of the samples in T1 (blue) and T2 (pink) treatments based on the pattern of gene expression. The color scale represents changes of gene expression: blue boxes represent genes downregulated and red and orange boxes represent genes upregulated.

Differential expression analyses showed 11 genes differentially expressed (DE) in liver between T1 and T2 piglets (Supplementary Table S1). Most of these DE genes, 9 out of 11, were upregulated in the T2 piglets under the high calorie regime (Fig. 1A). Histamine Receptor H1 (*HRH1*) was the most upregulated gene (FC = 20.77, *P*-value = 4.41 × 10^−06^), while 3-Hydroxy-3-Methylglutaryl-CoA Synthase 2 (*HMGCS2*) was the most downregulated gene in the T2 fed animals (FC^−1^ = 4.26, *P*-value = 2.40 × 10^−05^).

Regarding transcriptomic changes in adipose tissue, no DE genes were found in VAT, whereas 171 genes DE between T1 and T2 piglets were identified in SAT (Supplementary Table S2). Of these, 100 genes were upregulated and 71 were downregulated in the T2 fed animals (Fig. 1B,C) when compared to the control (T1) group. The genes most up- and downregulated in the T2 fed group were Myosin Heavy Chain 4 (*MYH4*; FC = 614.29, *P-*value = 8.72 × 10^−07^) and Iroquois Homeobox 1 (*IRX1*; FC^−1^ = 10.04, *P-*value = 1.47 × 10^−04^), respectively.

**Figure 2:**
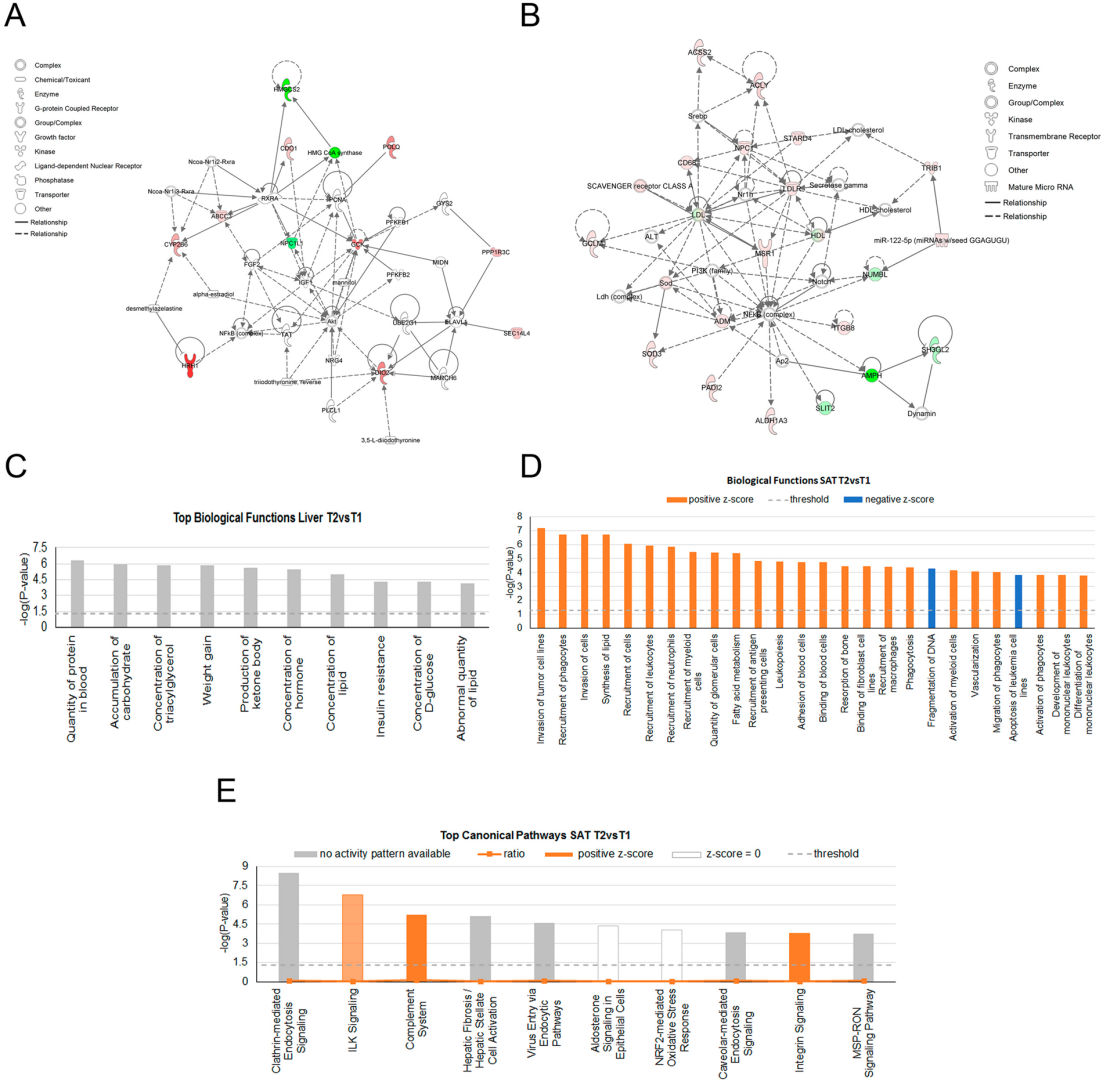
Functional categorization of differentially expressed (DE) genes in liver and subcutaneous adipose tissue (SAT) between T1 and T2 fed animals using the Core Analysis function included in the Ingenuity Pathway Analysis (IPA) software. Plots of the biological networks most significantly enriched by the list of DE genes between T1 and T2 fed animals in (**A**) liver: *Carbohydrate metabolism, Lipid metabolism and Molecular transport* and (**B**) SAT: *Lipid metabolism, Molecular transport and Small molecule biochemistry*. Red color indicates genes upregulated and green color downregulated in T2vsT1 groups. Top biological functions in liver (**C**) and activated biological functions in SAT (**D**) sorted by their *P*-values identified by the list of DE genes between T1 and T2 treatments. Threshold (grey horizontal dashed line) indicates the minimum significance level based on the Fisher exact test with a –log_10_ (*P*-value) > 1.3 (*P*-value < 0.05). Color indicates the activation state, orange for positive z-score > 2 (activation state increased in T2vsT1) and blue for negative z-score < −2 (activation state decreased in T2vsT1). E) Top canonical pathways identified by the list of DE genes in SAT between T1 and T2 treatments sorted by their *P*-values. Threshold (grey horizontal dashed line) indicates the minimum significance level based on the Fisher exact test with a –log_10_ (*P*-value) > 1.3 (*P*-value < 0.05). Ratio indicates the number of DE genes from the dataset that map to the pathway divided by the total number of genes that define the canonical pathway. Color indicates the activation state.

To gain a better understanding of the biological processes and pathways involved in the response to the high calorie diet, a functional annotation of DE genes in liver and SAT was performed. In liver, the only significant enriched network identified by IPA and containing all the DE genes between T1 and T2 was associated with carbohydrate metabolism, lipid metabolism and molecular transport (Fig. 2A). Accordingly, the biological functions most significantly enriched in the list of DE genes were related to the accumulation of carbohydrate, concentration of triacylglycerol, hormone, lipid and D-glucose, and production of ketone body among others (Fig. 2C). The full list of biological functions identified is shown in Supplementary Table S3. Finally, among the list of canonical pathways overrepresented, it is worth to highlight PXR/RXR activation with Cytochrome P450 Family 2 Subfamily B Member 6 (*CYP2B6*)*, HMGCS2*, and ATP Binding Cassette Subfamily C Member 3 (*ABCC3*) as components.

More noteworthy, we identified thirteen networks (Supplementary Table S4) significantly overrepresented in the set of DE genes in SAT. The top network was related with lipid metabolism, molecular transport and small molecule biochemistry (Fig. 2B). Furthermore, the top fourth network was associated with endocrine system development and function, lipid metabolism and small molecule biochemistry, with the Leptin (*LEP*) gene, upregulated in T2 compared with T1 (FC  =  1.81, *P*-value = 3.43 × 10^− 4^), taking part of most of the functions identified for this network. The list of biological functions annotated by IPA is shown in Supplementary Table S5. Among these biological functions, it is relevant to highlight several functions with a z-score greater than 2, which indicates an increase of the activation state of these biological functions in T2: recruitment of phagocytes (z-score = 2.820), synthesis of lipid (z-score = 2.002) and fatty acid metabolism (z-core = 2.273) (Fig. 2D). Finally, among the most significantly enriched canonical pathways in the list of DE genes in SAT (Supplementary Table S6), it is worth mentioning the clathrin-mediated endocytosis signaling, ILK signaling, complement system, aldosterone signaling in epithelial cells, NRF2-mediated oxidative stress response, insulin receptor signaling and AMPK signaling pathways (Fig. 2E).

### Upstream regulators of gene expression induced by a high calorie diet

Retinoid X Receptor Alpha (*RXRA*) and Peroxisome Proliferator Activated Receptor Alpha (*PPARA*) were the top upstream regulators identified in liver. *RXRA* directly interacts with six DE genes (Fig. 2A). *PPARA* heterodimerizes with the nuclear receptor *RXR* and plays a pivotal role in fatty acid catabolism^15^. In SAT, Sterol Regulatory Element Binding Transcription Factors 1 and 2 (*SREBP1* and *SREBP2*) were the top upstream transcription regulators. These transcription regulators are involved in sterol and fatty acid biosynthesis^16^. Remarkably, while in SAT SREBF1 (z-score = 2.097) and SREBF2 (z-score = 2.562) were activated in T2 compared to T1, PPARA in liver presented a negative z-score (−1.359).

### Transcriptomic changes produced in liver and adipose depots of animals fed a western-type diet supplemented with bioactive ingredients

The transcriptomes of liver, VAT and SAT of the two groups of piglets fed a western diet plus bioactive supplementation (T3 and T4; n = 5 each) were also analysed by RNAseq to study the effect at gene expression level of adding different bioactive ingredients in a high calorie diet. Together with *Bifidobacterium breve* probiotic supplementation, 50% of protein of animal origin in T2 was replaced by vegetal protein in T3 and T4 groups. Animals in T4 were also supplemented with omega-3 fatty acids.

**Figure 3:**
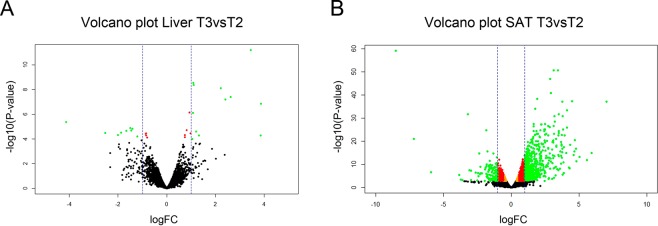
Volcano plots of differentially expressed (DE) genes between T2 and T3 fed animals in (**A**) liver and (**B**) subcutaneous adipose tissue (SAT). The vertical axis (y-axis) corresponds to the −log_10_ (*P*-value), and the horizontal axis (x-axis) displays the log_2_ fold change (logFC) value. Green dots represent DE genes according to a FDR < 0.05 in liver and FDR < 0.01 in SAT and |logFC| > 1. Red dots represent DE genes according to a FDR < 0.05 in liver and FDR < 0.01 in SAT and |logFC| > 0.58 and orange dots represent DE genes according to a FDR < 0.05 in liver and FDR < 0.01 in SAT. Positive x-values represent genes upregulated and negative x-values represent genes downregulated in T3vsT2 groups. The vertical dashed lines mark the |logFC| = 1.

A total of 30 genes were identified as DE in liver between T2 and T3 fed piglets, 17 upregulated and 13 downregulated in the T3 group when compared to the T2 group (Fig. 3A and Supplementary Table S7). Remarkably, the genes most up- and downregulated in T3 were Fibroblast Growth Factor 21 (*FGF21*; FC = **14.63**, *P*-value = 1.41 × 10^−07^) and Serine Dehydratase (*SDS*; FC^−1^ = **17.45**, *P*-value = 4.19 × 10^−06^), respectively.

In relation to adipose tissue, no genes DE between T2 and T3 groups were identified in VAT, similarly to previous T2vsT1 comparison. In SAT, the comparison between T2 and T3 diets reported the largest number of DE genes among all comparisons between dietary treatments, suggesting a vast effect of adding the probiotic plus the vegetal-origin protein on the SAT transcriptome. A total of 1818 DE genes were identified (Fig. 3B and Supplementary Table S8). Most of these DE genes, 1237 out of 1818, showed higher expression levels in T3 piglets, being DIRAS Family GTPase 3 (*DIRAS3*) the most upregulated gene in this group (FC = **129.93**, *P*-value = 7.02 × 10^−38^). In contrast, only 581 genes showed higher expression in T2 (vs T3) piglets, with three homeobox genes among the top upregulated genes in T2: Homeobox A10 (*HOXA10*; FC^−1^ = **364.69**, *P*-value = 8.04 × 10^−60^), Homeobox C10 (*HOXC10*; FC^−1^ = **147.0**, *P*-value = 1.05 × 10^−21^) and Homeobox A9 (*HOXA9*; FC^−1^ = **33.48**, *P*-value = 1.71×10^−113^).

**Figure 4:**
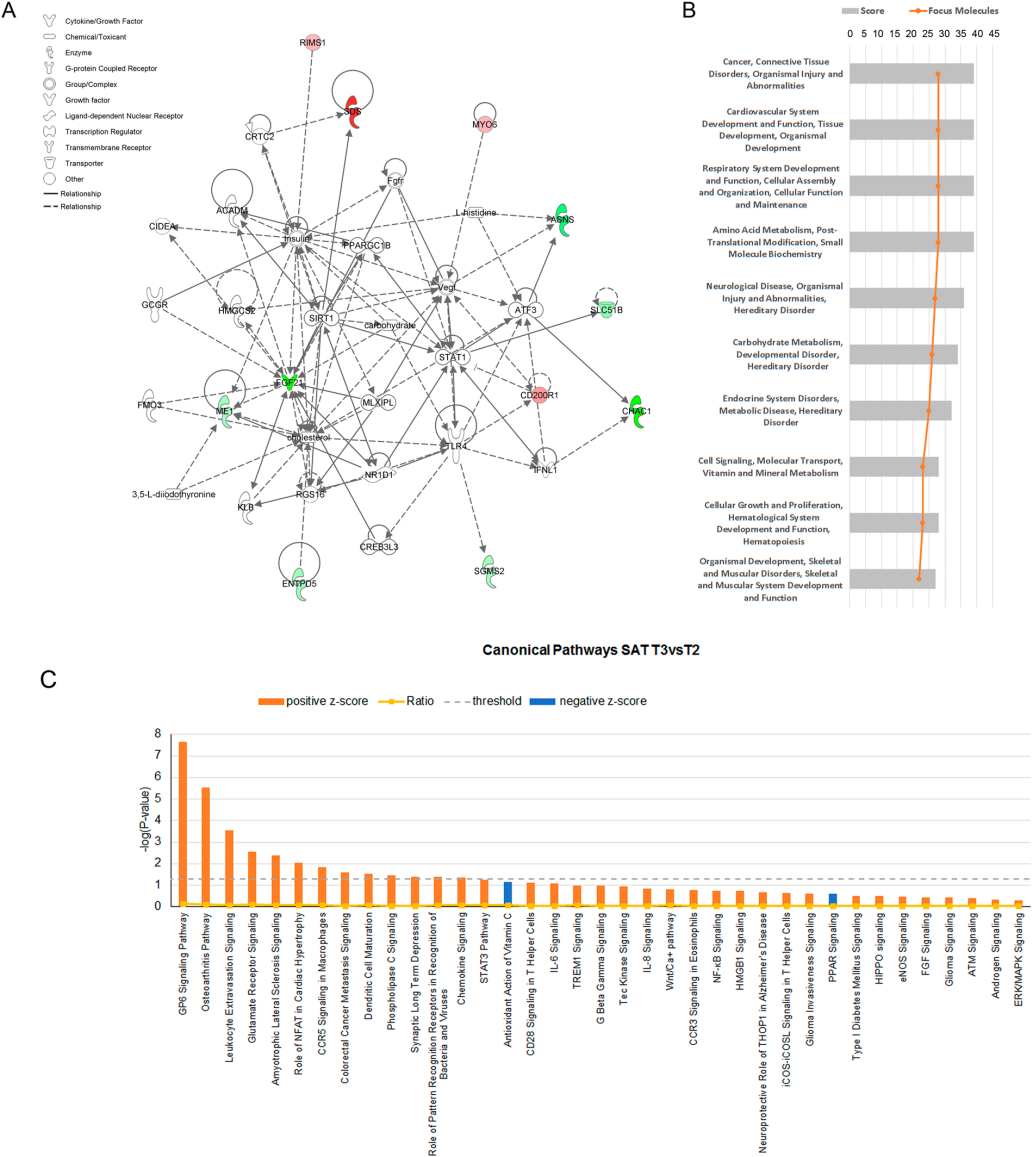
Functional categorization of differentially expressed (DE) genes in liver and subcutaneous adipose tissue (SAT) between T2 and T3 fed animals using the Core Analysis function included in the Ingenuity Pathway Analysis (IPA) software. (**A**) Plot of the biological network most significantly enriched by the list of DE genes between T2 and T3 fed animals in liver: *Carbohydrate metabolism, Molecular transport and Small molecule biochemistry*. Red color indicates genes downregulated and green color upregulated in T3vsT2 groups and (**B**) Top 10 biological networks most significantly enriched by the list of DE genes between T2 and T3 fed animals in SAT. The vertical axis (y-axis) corresponds to the diseases and functions enriched in the top ten networks. The horizontal axis (x-axis) displays the network score and the orange vertical line the focus molecules. (**C**) Canonical pathways sorted by their *P*-values identified by the list of DE genes between T2 and T3 treatments in SAT. Threshold indicates the minimum significance level based on the Fisher exact test with a −log_10_ (*P-*value) > 1.3 (*P*-value < 0.05). Ratio indicates the number of DE genes from the dataset that map to the pathway divided by the total number of genes that define the canonical pathway. Color indicates the activation state, orange for positive z-score > 2 (activation state increased in T3vsT2) and blue for negative z-score < −2 (activation state decreased in T3vsT2).

The functional annotation revealed that the top biological network enriched by the list of genes DE in liver was associated with carbohydrate metabolism, molecular transport and small molecule biochemistry (Fig. 4A). *FGF21* was central in this network, and was interconnected with molecules such as insulin, carbohydrate or cholesterol. The other network identified was associated with cellular movement, hematological system development and function, and immune cell trafficking. Finally, a significantly enriched group of DE genes (*ABCC3*, *EPHX1*, *SLC51B*) upregulated in the T3 group was identified as associated with the KEGG pathway bile secretion.

In SAT, a total of 25 networks were identified (Supplementary Table S9) as overrepresented in the list of DE genes. Among the top ten networks, it is noteworthy the presence of diseases and functions related to connective tissue disorders, amino acid metabolism, carbohydrate metabolism, endocrine system disorder, metabolic disease, cell signaling, and developmental processes (Fig. 4B). In addition, we identified a total of 35 canonical pathways with a z-score greater than 2 (activation state increased in T3 or decreased in T2) and two canonical pathways with a negative z-score lower than −2 (Fig. 4C and Supplementary Table S10). Among the canonical pathways with a positive z-core, it is worth to highlight GP6 signaling pathway, STAT3 pathway and IL-6, IL-8 and NF-kB signaling among others. The antioxidant action of vitamin C and PPAR signaling pathways have a negative z-score. Moreover, we identified with Cluego highly significant reactome pathways such as extracellular matrix organization, degradation of the extracellular matrix and collagen formation; GO biological processes such as blood vessel development and angiogenesis, and KEGG pathways such as PI3K-Akt signaling pathway, enriched with genes upregulated in the T3 group.

**Figure 5:**
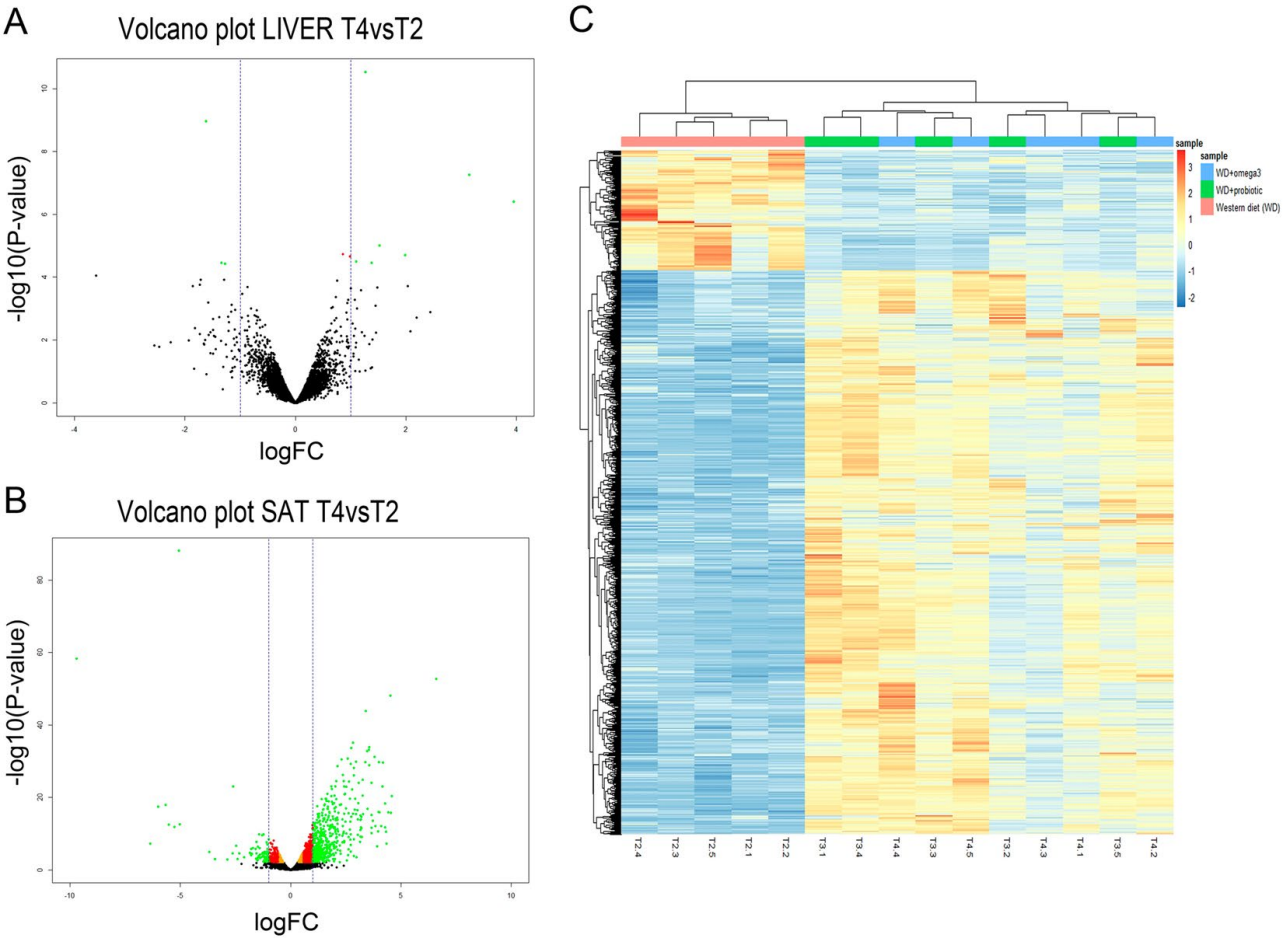
Differentially expressed (DE) genes in liver and subcutaneous adipose tissue (SAT) between T2 and T4 fed animals. Volcano plot displaying DE genes in liver (**A**) and SAT (**B**) between T2 and T4 treatments. The vertical axis (y-axis) corresponds to the -log_10_ (*P*-value), and the horizontal axis (x-axis) displays the log_2_ fold change (logFC) value. Green dots represent DE genes according to a FDR < 0.05 and |logFC| > 1. Red dots represent DE genes according to a FDR < 0.05 and |logFC| > 0.58 and orange dots represent DE genes according to a FDR < 0.05. Positive x-values represent genes upregulated and negative x-values represent genes downregulated in T4vsT2 groups. The vertical dashed lines mark the |logFC| = 1. (**C**) Heatmap of common differentially expressed genes found in SAT between T3vsT2 and T4vsT2 treatments. Each row represents a gene (n = 1167) and each column represents an individual (n = 15). Line graphs on top of the heatmap show clustering of the samples in T2 (pink) and between T3 and T4 (green and blue, respectively) treatments based on the pattern of gene expression. Note that the individuals of the T3 and T4 treatments were not clustered separately according to the treatment. The color scale represents changes of gene expression: blue boxes represent genes downregulated and red and orange boxes represent genes upregulated.

Comparison between T2 and T4 diets only revealed 12 DE genes in liver (Fig. 5A and Supplementary Table S11), many of which were common with those identified when comparing T2 and T3 diets, including *FGF21* (FC = **15.45**, *P*-value = 3.88 × 10^−07^).

Nor was any DE gene found in VAT, but we identified 1318 genes whose expression in SAT differed between T2 and T4 groups (Fig. 5B and Supplementary Table S12). As occurred in liver, most of these genes (1167 out of 1318) were also found to be DE in SAT between T2 and T3 (Fig. 5C).

As regards functional annotation of DE genes in liver, only one overrepresented pathway related to energy production, lipid metabolism and small molecule biochemistry was identified (Supplementary Fig. S2). In SAT, considering that there was a vast number of DE genes commonly identified in the T3vsT2 and T4vsT2 comparisons, it was not surprising to identify common networks at functional annotation level.

Given the similarity between transcriptomic changes observed in both T3vsT2 and T4vsT2 comparisons, additional DE analyses between T3 and T4 were performed to better assess changes in the expression profile derived from incorporating omega-3 fatty acids to the supplementation with probiotic and rice hydrolysate. Results showed a low impact on both liver and SAT transcriptomes, with only one gene identified as DE in each tissue (*PNPLA3* and *LCE3C* were downregulated in liver and SAT, respectively, of T4 fed animals).

### Upstream regulators of gene expression induced by bioactive ingredients

Activating Transcription Factor 4 (ATF4) was identified as upstream regulator of genes DE in liver. Remarkably, this transcriptional factor is activated in T3 compared to T2 (z-score = 1.986), regulating the expression of Asparagine Synthetase (Glutamine-Hydrolyzing) (*ASNS*), Activating Transcription Factor 5 (*ATF5*), ChaC Glutathione Specific Gamma-Glutamylcyclotransferase 1 (*CHAC1*) and *FGF21*. Also, we identified the Transcription Factor 7 Like 2 (TCF7L2) in the T3vsT2 comparison, with the following target genes: Ectonucleoside Triphosphate Diphosphohydrolase 5 (inactive) (*ENTPD5*), *FGF21*, Malic Enzyme 1 (*ME1*) and myosin VI (*MYO6*). TCF7L2 has been implicated in blood glucose homeostasis and in the type 2 diabetes disease risk^17^.

In SAT, the most significant upstream regulators identified were the Transforming Growth Factor Beta 1 (*TGFB1*; with the highest z-score > 6.5), Transforming Growth Factor Beta 2 (*TGFB2*; z-score > 3) and Transforming Growth Factor Beta 3 (*TGFB3*; z-score > 3.2), the latter also upregulated in both T3 and T4 piglets when compared with T2 animals. These growth factors inhibit adipogenesis and regulates ECM protein expression through the induction of collagens and fibronectin and the repression of MMPs^18,19^. In addition, another transcription factor that was found activated in T3 and T4 animals was the Signal Transducer and Activator of Transcription 3 (STAT3; z-score > 2). This transcription factor is key in the interleukin 10 (IL10) mediated anti-inflammatory response^20^.

### qPCR validation of RNA-seq data

Seven genes representatives of key pathways identified by the RNA-seq analysis were analysed by quantitative real-time PCR (qPCR) to validate the RNA-seq results: *ABCC3, GCK*, and *FGF21* genes in liver, and *COL2A1*, *DIRAS3*, *IL10* and *LEP* in SAT. When the pattern of gene expression levels was compared, high correlations were observed between qPCR and RNA-seq, ranging from 0.730 to 0.995 in liver and 0.609 to 0.986 in SAT, which confirmed a high reproducibility of the data. Furthermore, when the RQ mean values were compared between diets, most gene expression differences previously detected by RNA-seq were validated (Supplementary Fig. S3).

## Discussion

The development of effective strategies to prevent childhood obesity and its comorbidities requires a better understanding of this condition. The research of transcript-based biomarkers represents a powerful tool in the identification of mechanisms of relevance in the control of obesity^21–23^ and the impact exercised by different treatments.

The transcriptomes of metabolic relevant tissues from our porcine model of prepubertal obesity pointed that SAT was the tissue showing most relevant changes at transcriptomic level (largest number of DE genes) in all comparisons, whereas no changes in the expression profile of VAT were observed. Unlike VAT, SAT grows and differentiates very quickly from birth becoming the main fat depot in swine^24^. While SAT is used as a measure of general adiposity in pigs and contributes to overall metabolism, VAT accumulation has been associated with an increased pathological risk^25,26^. However, an independent role for SAT in insulin resistance has been suggested^27,28^, indicating that SAT is also a good predictor of metabolic risk and insulin resistance^29^. In keeping with these observations, the increase in adiposity after 10 weeks of high calorie intake was particularly relevant in the SAT depot, which could explain the transcriptional changes observed in SAT but not in VAT of animals fed with a western-type diet compared to animals fed a control diet.

The top networks involved in transcriptomic changes derived from a high-calorie diet in both liver and SAT tissues were related with carbohydrate metabolism, lipid metabolism, molecular transport, and small molecule biochemistry. Nonetheless, a network related with endocrine system development and function, in which the *LEP* gene was playing a relevant role, was identified in SAT. LEP is a circulating hormone produced by the adipose tissue that regulates energy homeostasis and whose overexpression has been described in different adipose depots of obese individuals^30^. In accordance, *LEP* gene was upregulated in the western-diet fed animals together with other genes (*ACLY* and *ACSS2*) related with an increase of lipid synthesis or fatty acid metabolism biological functions. Deletion of these genes in animal models protects against hepatic steatosis, fat deposition, obesity and attenuates atherosclerosis^31,32^. Other genes related with concentration and transport of cholesterol and sterol synthesis were also upregulated in animals fed a T2 diet (*ADM*, *LDLR*, *MSR1*, *NPC1*, *STARD4* and *TRIB1*). Dysregulation of most of these genes has been associated with fat deposition and obesity in different species^33–36^, being also involved in endothelial dysfunction and atherosclerosis^37,38^. On the contrary, we identified the Growth Factor Receptor Bound Protein 10 (*GRB10*) gene downregulated in the western-diet fed animals. GRB10 has been proposed as an important regulator of adipose tissue metabolism and energy homeostasis through the regulation of PI3K/Akt/mTORC1 signaling^39^. The fat specific disruption of this gene increased fat mass and obesity via suppressing mTORC1-mediated lipolysis in adipose tissues^39^.

Biological functions related with recruitment of macrophages and phagocytes but also leukocytes, neutrophils and myeloid cells were also activated in pigs on western diet. The development of atherosclerosis and insulin resistance in obesity has been directly linked to the activation of proinflammatory pathways in adipose tissue with macrophage infiltration contributing to this proinflammatory state^40,41^. In line with these results, we identified the CD68 molecule (*CD68*) gene upregulated in animals on the western diet. CD68 is a macrophage-associated antigen that correlated positively with adipocyte size and body mass in humans and presented high expression levels in human subcutaneous adipose tissue of obese individuals^42^. Furthermore, the gene *AGT* encoding angiotensinogen was upregulated in the western-diet fed group. Increased adipose angiotensinogen gene expression in obesity^43^ has been associated with the etiology of endothelial dysfunction^12^. Other genes (*CD163* and *LYZ*) related with inflammatory processes were also overexpressed in western-diet fed pigs. *LYZ* has been involved in the development of atherosclerosis in a juvenile obesity pig model^44^. Finally, it is worth mentioning the canonical pathway “complement system” composed of five molecules (*ITGB2*, *C4BPA*, *C1QC*, *C1QA*, *C1QB*) upregulated in animals on the western diet. Adipose expression of C1q complement was increased in obese individuals, suggesting that classic pathway activation may play an important role in adipose tissue inflammation and insulin resistance^45^. The Complement C3 (*C3*) gene was also among the genes upregulated in the western-diet fed group. The increased production of C3 in adipose tissue may lead to both increased infiltration of macrophages and stimulated triglyceride synthesis in adipose tissue, thus contributing to insulin resistance and atherosclerosis^46,47^.

In liver, four genes related with the metabolism of carbohydrate function were upregulated in western-fed animals (*HRH1*, *GCK*, *DIO2* and *PPP1R3C*) compared with control ones. A metabolic crosstalk between glycogen and lipogenesis was demonstrated in mice fed with a HFD; the overexpression of Protein Phosphatase 1 Regulatory Subunit 3C (*PPP1R3C*, also known as *PTG*) dramatically augmented the hepatic accumulation of glycogen which shifted energy storage from glycogen to lipid through the increased expression of lipogenic genes^48^. Furthermore, the product of *DIO2* gene activates thyroid hormone (TH) which increases fatty acid uptake in the liver and hepatic lipogenesis^49^. Some authors suggest that TH may promote the synthesis of HDL^49^, which in turn may explain the high HDL cholesterol levels in serum of western-fed pigs.

On the other hand, the most downregulated gene in the liver of animals fed a high-calorie diet was *HMGCS2*. The protein codified by this gene is a mitochondrial enzyme that catalyses the first reaction of ketogenesis to provide with energy extrahepatic tissues in periods of carbohydrate deprivation such as fasting^50^. The higher levels of energy supplied to pigs fed a western-type diet (3621 kcal/kg) compared with pigs fed a control diet (2587 kcal/kg) suggest that, in the first group, the energy is directed towards the metabolic pathways of glycogenesis, *de novo* lipogenesis and cholesterogenesis with a decrease in ketogenesis.

Switches in the liver expression profile derived from feeding an HFD affected also genes related with whole-body cholesterol homeostasis. In physiological conditions, cholesterol is excreted by the liver either by secreting it into bile or by converting it into bile acids, the end products of cholesterol catabolism^51,52^. To this regard, we identified as upregulated in the western-fed animals different genes related with the conversion of cholesterol to bile acids (*CYP2B6*), taurine conjugation of bile acids (*CDO1*) and export of bile acids from hepatocytes (*ABCC3*). Additionally, the NPC1 Like Intracellular Cholesterol Transporter 1 (*NPC1L1*) gene was downregulated in this western-diet fed group. NPC1L1 localizes to the canalicular membrane of hepatocytes and facilitates the biliary cholesterol re-uptake into hepatocytes^53^. These results suggest an increased biliary cholesterol secretion and cholesterol catabolism in animals fed a western-type diet that may explain the lack of differences between cholesterol concentrations between T2 and T1 fed groups and the increased faecal fat content observed in the animals on the western-type diet (T1 = 19.5 ± 1.3 mg/g vs T2 = 50 ± 8 mg/g).

Taken together, our findings suggest an increased lipogenesis, cholesterogenesis and decreased lipolysis in animals on the western diet, which contributed to raise fat content and triggered inflammatory processes through the activation of the complement system and the recruitment of immune cells such as macrophages. All these processes could be involved in the development of endothelial dysfunction and insulin resistance and later on could trigger metabolic syndrome.

After studying the transcriptional changes produced in our pig model of diet-induced prepubertal obesity, we examined the effect of adding different bioactive ingredients to animals under a western-type diet. At phenotypic level, animals supplemented with *Bifidobacterium breve* probiotic and rice hydrolysate (with or without omega-3 PUFA) showed lower body weight gain and a suggestive tendency to lower fat content than no supplemented (T2) pigs feeding the same HFD. Similar results have been previously reported in other HFD-animal models supplemented with similar bioactive components^2,3,6–8^. The supplementation with *Bifidobacterium breve* probiotic plus rice hydrolysate did not improve significantly the serum levels of total cholesterol and triglycerides, but pigs additionally supplemented with omega 3 showed a decrease in the serum levels of LDL.

These phenotypic differences were also accompanied by changes in the transcriptional profiles of liver and SAT tissues of the supplemented animals, with the largest number of DE genes observed in animals fed with the probiotic *Bifidobacterium breve* plus rice hydrolysate (diet T3). The addition of omega 3 supplementation (T4vsT3) produced a negligible variation in the expression profile of both liver and SAT, which suggests that incorporating omega-3 fatty acids beyond the *Bifidobacterium breve* probiotic and rice hydrolysate supplementation has a low modulation effect on both liver and SAT transcriptomes. Considering these results, in the following section we only discuss the role of DE genes and biological processes involved in the response to the supplementation with *Bifidobacterium breve* probiotic and rice hydrolysate. In addition, since the T3 and T4 treatments contain more than one bioactive ingredient, further analysis is needed to elucidate whether transcriptional changes occur due to the specific effect of an ingredient or the interaction among them.

Supplementation with bioactive ingredients produced a high impact on the transcriptomic profile of SAT, where we identified the greatest number of DE genes and functions when comparing supplemented vs non-supplemented HFD early obese piglets. The most upregulated gene was the *DIRAS3* gene, whose expression levels increased till ~130 folds in animals receiving *Bifidobacterium breve* probiotic plus rice hydrolysate. This gene has been identified as upregulated in adipose-derived stromal/progenitor cells in SAT of long-term weight losing initially obese human females, in which *DIRAS3* gene reduced adipogenesis and activated autophagy through the negative regulation of Akt-mTOR signalling^54^. In line with this result, genes involved in the induction of adipogenesis pathway^55^ (*BMP5*, *BMP7*, *CEBPA* and *ZNF521*) were identified (either up- or down-regulated) in animals fed on western diet while genes (*BMP2* and *RUNX2*) that favour MSCs differentiation into osteoblasts vs adipogenic commitment^56^ resulted upregulated in the supplemented pigs.

We observed also an overrepresentation of pathways related with vascular and ECM remodelling: GP6 signalling pathway, extracellular matrix organization, degradation of extracellular matrix and collagen formation, blood vessel development and angiogenesis. These pathways gather genes, upregulated in supplemented animals, that encode collagen proteins (COL2A1, COL8A2, COL11A2, COL8A1, COL11A1, COL12A1, COL1A2, COL1A1, COL21A1, COL16A1, COL28A1, COL6A6, COL14A1, COL6A3, COL6A5, COL6A2, COL6A1, COL4A5, COL3A1, COL5A1) and other ECM components (CYR61, CTGF, MMP2, MMP16, MMP17, NOV, SPARC, TIMP1, TIMP2, THBS1, THBS2, THBS3 and THBS4). Furthermore, angiogenic factors such as VEGFD or ANGPTLs (ANGPTL1, ANGPTL2, ANGPTL5) growth factors were upregulated in the supplemented dietary treatments.

Although over-expression of proteins related to ECM remodelling has been associated with exacerbated fibrosis and metabolic alterations in obese individuals, there are also studies supporting beneficial effects of adipose tissue fibrosis^19,55^. In fact, up-regulation of ECM components has been also observed in individuals after bariatric surgery^57,58^ or experiencing weight loss during very low-calorie diet^29^. Therefore, the reduction in adipose accumulation observed in animals supplemented with *Bifidobacterium breve* probiotic plus rice hydrolysate may be accompanied by intense extracellular matrix remodelling to favour adequate plasticity and expandability of adipose tissue. However, histopathology studies are needed to better understand the role of ECM remodelling in adipose tissue of animals supplemented with *Bifidobacterium breve* and rice hydrolysate.

Weight loss in obese subjects has been also associated with decreased expression of inflammatory markers joined with the concomitant up-regulation of anti-inflammatory cytokines such as Interleukin 10 (IL10)^29^. IL10 is a major immunosuppressive cytokine, and together with STAT3 is essential for the anti-inflammatory response^20^. Transgenic mice with muscle-specific overexpression of IL10 were more insulin sensitive and protected from HFD-induced inflammatory response in muscle^59^. Here, we found the *IL10* gene and its receptor (*IL10RA*) upregulated in HFD piglets receiving the T3 diet, probably indicating an improvement of the inflammatory profile of SAT in obese animals derived from the ingestion of these bioactive ingredients. In close support with our view, other genes with anti-inflammatory effects such as TNF alpha induced protein 6 (*TNFAIP6*)^60^ and TNF alpha induced protein 3 (*TNFAIP3*)^61^ were also identified as upregulated in supplemented piglets.

Finally, it is worth noting the huge down-regulation of several homeobox genes (particularly *HOXA10* and *HOXC10*) in SAT derived from bioactive supplementation. A cohort of recent studies have demonstrated a role of HOX genes in the regulation of body fat distribution and the origin of obesity (reviewed in^62^). However, higher expression of HOX genes have been shown in lean individuals compared to obese people^62^ or in individuals after a bariatric surgery^58^; although it is unclear the mechanisms underlying these expression changes.

The gene most upregulated in the liver of piglets supplemented with *Bifidobacterium breve* and rice hydrolysate was the *FGF21* gene, whose expression levels were ~15 folds higher in these animals than in western-fed but no supplemented animals (T2). Remarkably, this gene has been extensively reported to counteract obesity and its comorbidities in many animal models^63,64^, emerging as a promising therapeutic agent for the treatment of obesity and insulin resistance^65^. FGF21 is a peptide hormone and is primarily secreted by the liver. In this tissue *FGF21* gene functions as a starvation signal inducing gluconeogenesis, fat oxidation and ketogenesis^66^. The up-regulation of *FGF21* was accompanied by the up-regulation of *ATF5* and *ASNS*, all genes regulated by the transcription factor ATF4. It is worth mentioning that similar results have previously been observed in a hepatic knockout mouse for *CREP* (also known as Protein Phosphatase 1 Regulatory Subunit 15B). This gene is a subunit of the eIF2α phosphatase complex, a central component of the integrated stress response (ISR) signalling. Its hepatic knockout leads to upregulated activity of the downstream ISR signalling protein, ATF4 and its target genes *ASNS*, *ATF5* and *FGF21*. Additional results based on further knockout mice models suggested that liver activation of IRS may be a viable target to treat obesity through the upregulation of *FGF21*^67,68^. Finally, three genes associated with lipid and bile acid secretion (*ABCC3*, *EPHX1*, *SLC51B*) were upregulated in T3 fed pigs when compared to animals on the non-supplemented western-type diet. Up-regulation of Solute Carrier Family 51 Beta Subunit (*SLC51B*; also called *OSTβ*) in human and rodent livers has been associated with a protective role under cholestatic conditions^69^.

Taken together, our findings suggest an increased anti-inflammatory response, vascular and ECM remodelling and decreased lipogenesis in SAT of animals supplemented with *Bifidobacterium breve* and rice hydrolysate. Furthermore, our results are in accordance with the induction of fatty acid oxidation and excretion of faecal lipid, total cholesterol and bile acid in liver, as previously described in rodent models fed HFD and supplemented with either hydrolysates of rice protein^7^ or *Bifidobacterium breve* B-3^4^.

**Figure 6:**
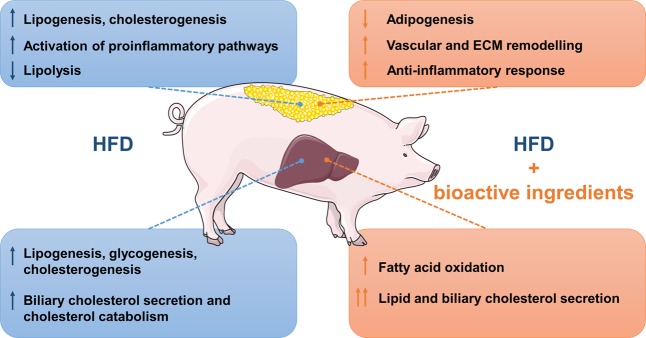
Overview of the main metabolic pathways up and down-regulated in liver and subcutaneous adipose tissue (SAT) of HFD-induced obese piglets supplemented with *Bifidobacterium breve* and rice hydrolysate. This figure was created using images provided by Smart Servier Medical Art.

In conclusion, the present study demonstrates that the western-type diet induces changes in the expression of genes related to lipid and cholesterol metabolism, immunity and inflammation in our pig model of prepubertal obesity. In addition, our findings show that the supplementation of HFD-induced obese piglets with different bioactive ingredients (*Bifidobacterium breve* and rice hydrolysate) modulates genes and pathways implicated in catabolic processes, cholesterol metabolism, adipogenesis, inflammation and ECM remodelling (Fig. 6). These results corroborate some of the previously published results in murine models of obesity supplemented with these bioactive ingredients, and shed new light in the molecular mechanisms modified by them in the liver and SAT tissues of HFD-induced obese pigs. Finally, we have identified DE genes that might be used as biomarkers to improve childhood obesity and its associated comorbidities.

## Methods

### Experimental device (animals, treatments, growth monitoring and biochemical analyses)

Animals used for this study belonged to a high intramuscular fat commercial Duroc pig line. The experiment was carried out with 48 contemporary female piglets born in 12 different litters (i.e. 12 groups of 4 littermates). After weaning (~4 weeks of age), these 48 piglets were transferred to the IRTA pig experimental farm in Monells (Girona, Spain), housed in transition devices and fed *ad libitum* a standard transition diet. At ~9 weeks of age animals were assigned to four dietary treatments (10 to 11 animals per treatment) and distributed in four different pens located in environmentally monitored facilities. Each sibling from a litter was assigned to a different treatment, so that piglets in one pen were full sibs of piglets in other pen. During the following 10 weeks animals were fed *ad libitum* four different diets (T1 to T4): T1) a conventional (and balanced) growth diet according to NRC (Nutrition Resource Centre) recommendations; T2) a western-type diet formulated with a high fat content and protein of animal origin (caseinate); T3) a western-type diet in which 50% of the protein was substituted by protein of vegetal origin (rice hydrolysate) and soft cheese including 5 × 10^10^ cfu/day *Bifidobacterium breve* probiotic (CECT8242); and T4) similar to T3 but adding omega-3 fatty acids to the diet (1 g stearidonic acid and 2 g docosahexaenoic acid per 100 g fat). Experimental diets were elaborated at the IRTA experimental mill in Mas de Bover (Tarragona, Spain); details about components and nutritive composition of diets are reported in Supplementary Tables S13 and S14, respectively.

For growth monitoring, all pigs were weighed individually at the beginning and every two weeks during the whole experiment, plus the day before slaughtering. Individual feed intake was also recorded by means of electronic feeders located in each pen (IVO-station feeder; INSENTEC®, The Netherlands). Computed tomography (CT) was used to obtain one axial image at the level of the 2nd lumbar vertebrae from all pigs at ~18 weeks of age. The CT-scanning of pigs was performed under anesthesia and after 16 h fasting, using the General Electric HiSpeed ZX/I (General Electric, Fairfield, CN, USA) equipment. Image analyses were performed with the software VisualPork. Image processing allowed transforming the areas of interest into volumes (mm^3^) of different fat depots according to^70^, thus obtaining estimates of the relative fat volume and the relative amount of subcutaneous, intermuscular and flare fat.

When animals reached 19 weeks of age, they were slaughtered at IRTA experimental slaughterhouse in totally controlled conditions and in compliance with all welfare regulations. Blood samples for biochemical analyses were taken from overnight fasted animals immediately before sacrifice. After serum separation by centrifugation, lipids and other conventional biochemical variables, including total cholesterol, LDL cholesterol, HDL cholesterol, triglycerides and glucose, were measured by using commercial kits from Spinreact (Girona, Spain). Samples of approximately 1 g from liver, visceral adipose tissue (VAT) and subcutaneous adipose tissue (SAT) were collected immediately after slaughter, submerged in RNA-later (Sigma, Spain) and stored at −80 °C after 24 h, according to the protocols recommended by the manufacturer.

All experimental procedures with pigs, including management, anesthetizing, monitoring, blood sampling and slaughtering, were performed according to the Spanish Policy for Animal Protection RD1201/05, which meets the European Union Directive 86/609 about the protection of animals used in experimentation. The experimental protocol was approved by the Ethical Committee of the Institut de Recerca i Tecnologia Agroalimentàries (IRTA).

Finally, the dietary treatment effects on piglet’s weight, growth, feed/calorie intake, fat deposition and biochemical variables were assessed by a one-way analysis of variance, and differences between the corresponding least square means were tested by a Tukey’s HSD test for multiple comparisons.

### mRNA sequencing and RNA-seq data analysis

Total RNA was isolated from liver (n = 20, five animals per treatment), visceral adipose tissue (VAT; n = 20) and subcutaneous adipose tissue (SAT; n = 20) samples using the Ambion RiboPure (Thermo Fisher Scientific). RNA was quantified in a Nanodrop ND-1000 spectrophotometer and RNA purity and integrity was checked by using a Bioanalyzer-2100 equipment (Agilent Technologies, INC., Santa Clara, CA). Libraries were prepared using the TruSeq RNA Sample Preparation Kit (Ilumina Inc., CA) and were paired-end sequenced (2 × 75 bp), by using the TruSeq SBS Kit v3-HS (Illumina Inc., CA), in a HiSeq. 2000 platform (Illumina Inc., CA). More than 30 M PE reads were obtained for all samples. All sequencing tasks were carried out in the Centro Nacional de Análisis Genómico (Barcelona, Spain).

The quality of the raw sequenced reads in the FASTQ files was analysed with the FASTQC software (Babraham Bioinformatics, http://www.bioinformatics.babraham.ac.uk/projects/fastqc/). Reads were mapped to the reference pig genome Sscrofa11.1 and the annotation database Ensembl Genes 92 (http://www.ensembl.org/info/data/ftp/index.html) by using STAR v. 2.5.3a^71^. Transcript quantification was performed with RSEM v. 1.3.0^72^. The R package EdgeR^73^ was used to identify differentially expressed (DE) genes based on the RNA-seq data. Genes with a Fold Change (FC) above 1.5 (i.e. |log_2_FC| > 0.58) and FDR < 0.05 after correcting for multiple testing were classified as DE.

The R package pheatmap and the R VolcanoPlot function were used to graphically represent the expression level and significance of DE genes among treatments.

### Functional analysis

Prior to perform functional analyses, orthologous human gene names, from the list of DE genes with a FC above 2 (i.e. |log_2_FC| > 1) and FDR < 0.05, were retrieved from the Ensembl Genes 92 Database using the Biomart software^74^. ClueGO v. 2.3.5 plug-in of Cytoscape v. 3.2.1^75^ and the Core Analysis function included in the Ingenuity Pathway Analysis software were used to obtain gene ontologies (GO), biological functions, gene networks and canonical pathways significantly (padj < 0.05) enriched in the set of DE genes. Activation z-scores greater than 2 or lower than −2 were considered to infer the activation states (increased and decreased, respectively) of functional annotations.

### Validation of differentially expressed genes by RT-qPCR

A quantitative real-time PCR (qPCR) assay using SYBR Green chemistry (SYBR^TM^ Select Master Mix, Applied Biosystems) was performed to validate the results obtained by RNA-Seq. The isolated RNA of individual samples for liver and SAT tissues from T1, T2, T3 and T4 treatments was reverse-transcribed into cDNA using the PrimeScript RT Reagent Kit (TAKARA) in a total volume of 20 μl containing 1 μg for liver or 500 ng for SAT of total RNA, following the manufacturer’s instructions. All target-primers, except for the *IL10* gene^76^, were designed using PrimerExpress 2.0 software (Applied Biosystems) and are shown in Supplementary Table S15. The *ACTB* and *HPRT1* genes were used as endogenous controls^77^. All assays were tested and PCR efficiencies were evaluated by performing standard curves with a four-fold dilutions series (1/4, 1/16, 1/64, 1/256, 1/1024) per triplicate of a pool of 5 cDNA samples in an Applied Biosystems 7500 Real-Time PCR System (Applied Biosystems, Inc.; Foster City, CA). A dissociation curve was drawn for each primer pair to assess for the specificity of the reactions. A QuantStudio™ 12 K Flex Real-Time PCR System (Thermo Fisher Scientific) was used for mRNA quantification. The reactions were carried out in a 384-well plate in 15 μl volume containing 3.75 μl of cDNA sample diluted 1/20. Primer concentration is reported in Supplementary Table S15. The thermal cycle was: 10 min at 95 °C, 40 cycles of 15 sec at 95 °C and 1 min at 60 °C. Each sample was analysed in duplicate. Data was analysed using the Thermo Fisher Cloud software (Applied Biosystems) and the comparative Ct method^78^. A sample from an animal fed with the western-diet (T2) was selected as calibrator. Correlation coefficients between RNA-seq and qPCR data (RQ) as well as group mean comparison test were computed with R.

## Supplementary information

Supplementary information.

Supplementary information2.

Supplementary information3.

Supplementary information4.

Supplementary information5.

Supplementary information6.

Supplementary information7.

Supplementary information8.

Supplementary information9.

Supplementary information10.

Supplementary information11.

Supplementary information12.

Supplementary information13.

Supplementary information14.

Supplementary information15.

Supplementary information16.

## Data Availability

Data will be available in Sequence Read Archive (SRA). Submission number: PRJNA577556.
