# Supplementary material for: Dietary intake of bioactive ingredients impacts liver and adipose tissue transcriptomes in a porcine model of prepubertal early obesity
Source: Sci Rep. 2020 Mar 25;10:5375. doi: 10.1038/s41598-020-62320-4 (PMC7096439; doi:10.1038/s41598-020-62320-4)
Supplement: Supplementary file 1 — Supplementary information. [file 41598_2020_62320_MOESM1_ESM.docx]

**SUPPLEMENTARY MATERIAL**

**Dietary intake of bioactive ingredients impacts liver and adipose tissue transcriptomes in a porcine model of prepubertal early obesity**

Maria Ballester^1^*, Raquel Quintanilla^1^, Francisco J. Ortega^2,3^, José C.E. Serrano^4^, Anna Cassanyé^4^, Maria Rodríguez-Palmero^5^, José A. Moreno-Muñoz^5^, Manuel Portero-Otin^4^, Joan Tibau^6^

^1^Animal Breeding and Genetics Programme, Institute for Research and Technology in Food and Agriculture (IRTA), Torre Marimon, 08140, Caldes de Montbui, Spain.

^2^Department of Diabetes, Endocrinology, and Nutrition (UDEN), Institut d'Investigació Biomèdica de Girona (IdIBGi), Girona, Spain.

^3^Centro de Investigación Biomédica en Red de la Fisiopatología de la Obesidad y la Nutrición (CIBEROBN), Instituto de Salud Carlos III (ISCIII), Madrid, Spain.

^4^Department of Experimental Medicine, University of Lleida-Biomedical Research Institute of Lleida, 25196 Lleida, Spain

^5^Basic Research Department. Ordesa Laboratories, 08830 Barcelona, Spain.

^6^Animal Breeding and Genetics Programme, Institute for Research and Technology in Food and Agriculture (IRTA), Finca Camps i Armet, 17121, Monells, Spain.

*E-mail: maria.ballester@irta.cat

**Figure S1.** PCA plot of RNA-seq data from SAT samples (n = 20) showing an outlier in the control group (T1, sample AD5656). Samples from each treatment are indicated by different colours (Red: T1; Green: T2; Blue: T3; Violet: T4).


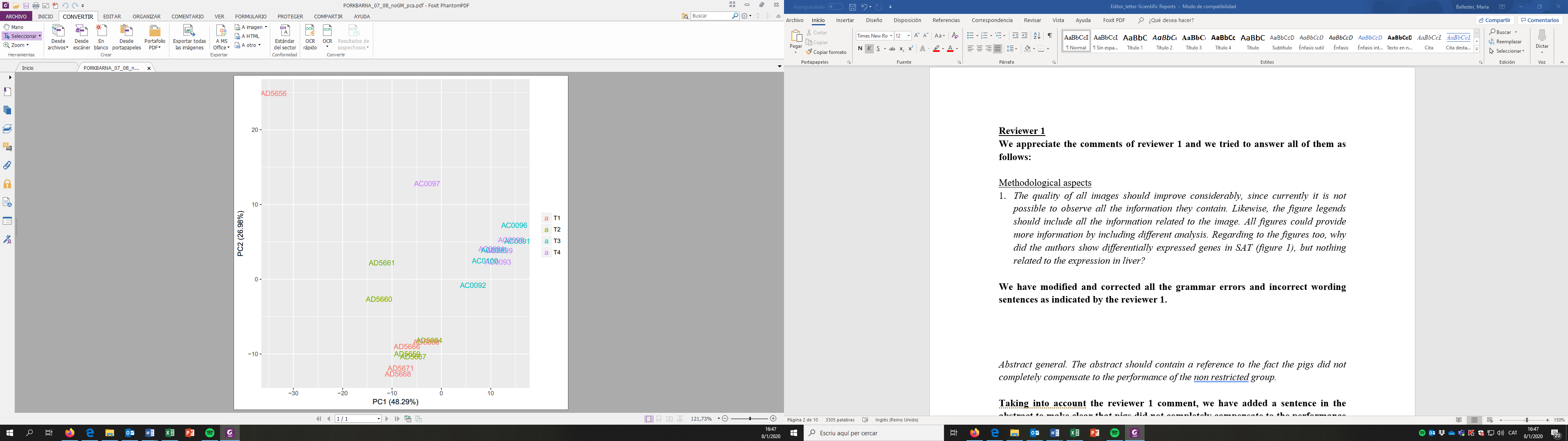


**Figure S2.** Plot of the biological network most significantly enriched by the list of genes differently expressed between T2 and T4 fed animals in liver: *Energy production, Lipid metabolism and Small molecule biochemistry*. Red color indicates genes downregulated and green color upregulated in T4vsT2 groups.


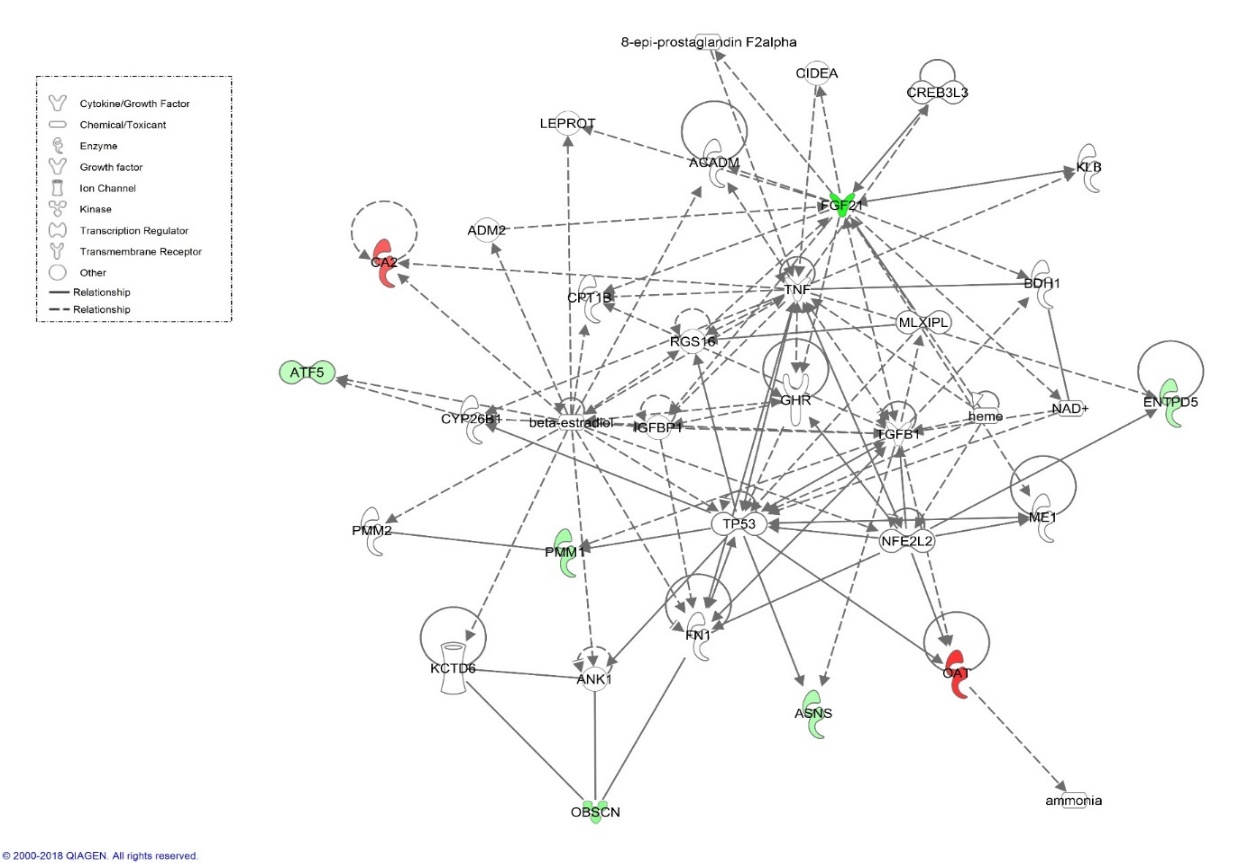


**Figure S3.** Plots showing mean gene expression levels (RQ) obtained in the validation by qPCR for ABCC3 (A), GCK (B), and FGF21 (C) in liver and LEP (D), DIRAS3 (E), COL2A1 (F), and IL10 (G) in SAT according to different diets. Data represents means ± SEM. Values with different superscript letter (a and b) indicate significant differences between groups (*P*-value ≤ 0.05). Correlation values between qPCR and RNA-seq expression data are indicated below each corresponding panel.

R_qPCRvsRNA-seq_= 0.730

R_qPCRvsRNA-seq_= 0.995

R_qPCRvsRNA-seq_= 0.933

R_qPCRvsRNA-seq_= 0.760

R_qPCRvsRNA-seq_= 0.986

R_qPCRvsRNA-seq_= 0.986

R_qPCRvsRNA-seq_= 0.609

A

B

C

D

E

F

G


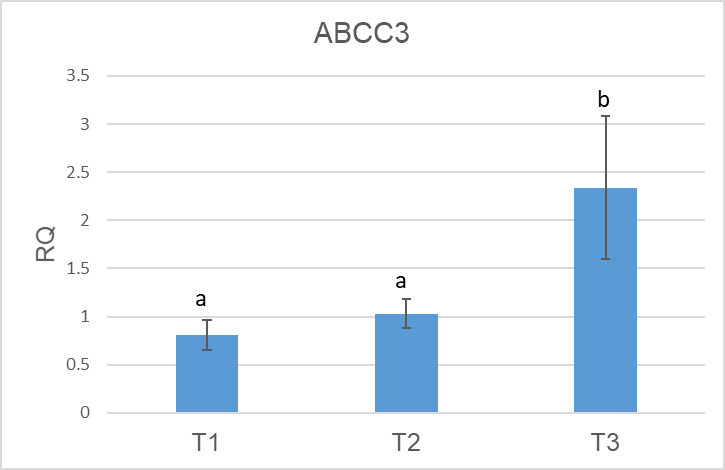

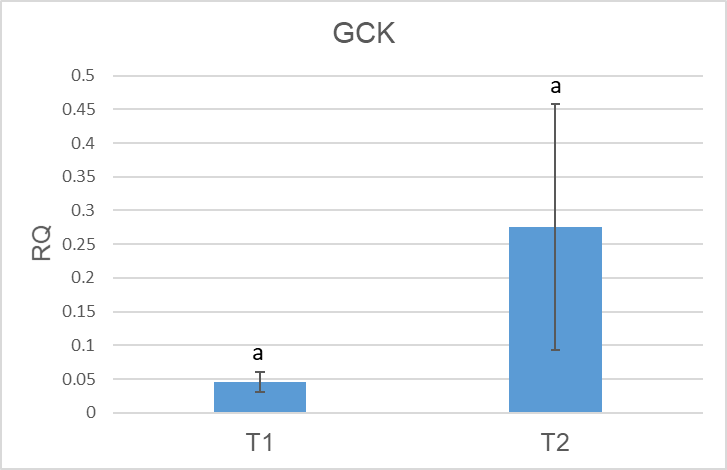

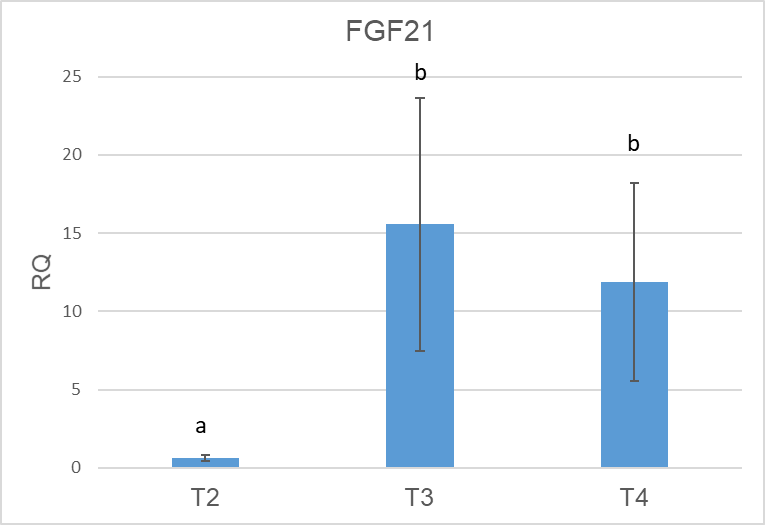

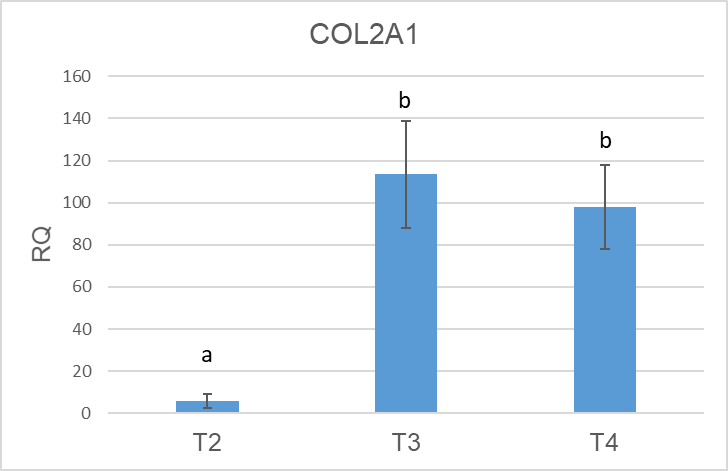

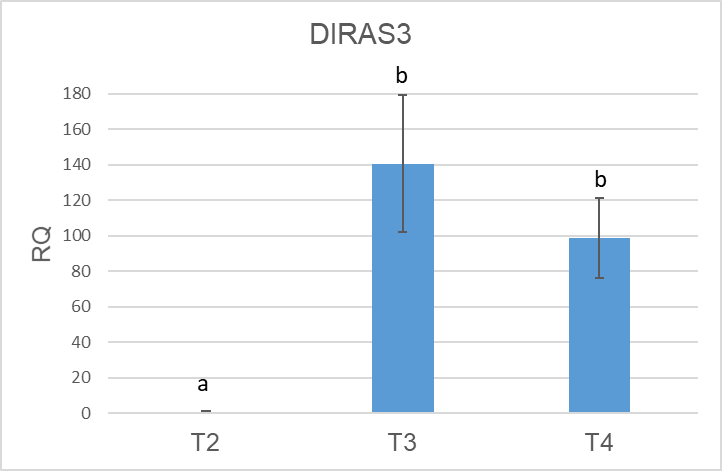

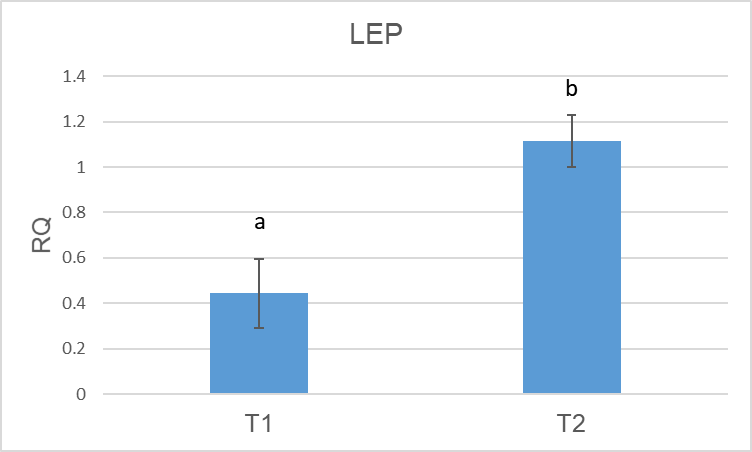

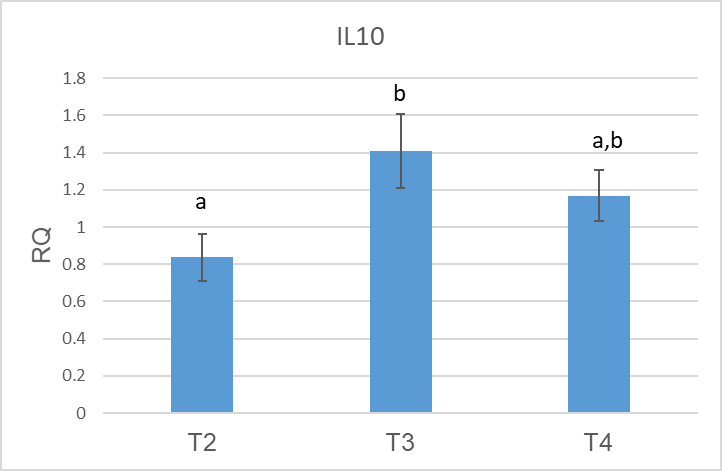


**Table S1.** List of genes differentially expressed in liver between T1 and T2 fed pigs considering an FDR<0.05 and FC>1.5 (i.e ǀlogFCǀ > 0.58).

**Table S2.** List of genes differentially expressed in subcutaneous adipose tissue (SAT) between T1 and T2 fed pigs considering an FDR<0.05 and FC>1.5 (i.e ǀlogFCǀ > 0.58).

**Table S3.** Biological functions identified in the list of differently expressed genes (FDR<0.05 and FC>2 (i.e ǀlogFCǀ>1)) in liver between animals fed with T1 and T2 diets.

**Table S4.** List of networks enriched by genes differentially expressed (FDR<0.05 and FC>2 (i.e ǀlogFCǀ>1)) in subcutaneous adipose tissue (SAT) between T1 and T2 fed pigs.

**Table S5.** Biological functions identified in the list of differently expressed genes (FDR<0.05 and FC>2 (i.e ǀlogFCǀ>1)) in subcutaneous adipose tissue (SAT) between animals fed with T1 and T2 diets.

**Table S6.** List of canonical pathways identified in the list of differently expressed genes (FDR<0.05 and FC>2 (i.e ǀlogFCǀ>1)) in subcutaneous adipose tissue (SAT) between animals fed with T1 and T2 diets.

**Table S7.** List of genes differentially expressed in liver between T2 and T3 fed pigs considering an FDR<0.05 and FC>1.5 (i.e ǀlogFCǀ > 0.58).

**Table S8.** List of genes differentially expressed in subcutaneous adipose tissue (SAT) between T2 and T3 fed pigs considering an FDR<0.05 and FC>1.5 (i.e ǀlogFCǀ > 0.58).

**Table S9.** List of networks enriched by genes differentially expressed (FDR<0.05 and FC>2 (i.e ǀlogFCǀ>1)) in subcutaneous adipose tissue (SAT) between T2 and T3 fed pigs.

**Table S10.** List of canonical pathways with a z-score ± 2 identified in the list of differently expressed genes (FDR<0.05 and FC>2 (i.e ǀlogFCǀ>1)) in subcutaneous adipose tissue (SAT) between animals fed with T2 and T3 diets.

**Table S11.** List of genes differentially expressed in liver between T2 and T4 fed pigs considering an FDR<0.05 and FC>1.5 (i.e ǀlogFCǀ > 0.58).

**Table S12.** List of genes differentially expressed in subcutaneous adipose tissue (SAT) between T2 and T4 fed pigs considering an FDR<0.05 and FC>1.5 (i.e ǀlogFCǀ > 0.58).

**Table S13.** Components of experimental diets T1 to T4.

**Table S14.** Nutritive composition of experimental diets.

**Table S15.** List of primers for the qPCR analysis.
